# Monoarticular synovitis of knee: dealing with the dilemma

**DOI:** 10.1051/sicotj/2020044

**Published:** 2020-12-11

**Authors:** Tarun Goyal, Souvik Paul, Arghya Kundu Choudhury, Tushar Kalonia

**Affiliations:** 1 Additional Professor, Department of Orthopaedics, All India Institute of Medical Sciences Virbhadra Marg 248201 Rishikesh India; 2 Senior Resident (Academic), Department of Orthopaedics, All India Institute of Medical Sciences 248201 Rishikesh India; 3 Junior Resident, Department of Orthopaedics, All India Institute of Medical Sciences 248201 Rishikesh India; 4 Senior Resident, Department of Pathology, All India Institute of Medical Sciences 248201 Rishikesh India

**Keywords:** Knee, Monoarticular synovitis, Synovial biopsy, Chronic synovitis, Synovectomy, Level IV, Observational study

## Abstract

*Introduction*: Chronic synovitis involving a single large joint remains a diagnostic dilemma. We present 61 cases of chronic synovitis of the knee, followed prospectively for 2 years. The study focuses on the diagnosis, management, and histopathological correlation. *Methods*: We prospectively studied 61 patients with chronic mono-articular synovitis of the knee joint, between July 2016 and September 2017. All patients underwent plain radiographs, magnetic resonance imaging, and arthroscopic examination with synovial biopsy. Further treatment was based on findings of histopathological examination. *Results*: The average duration of symptoms was 7.72 ± 4.34 months. The mean age at presentation was 29.93 ± 15.56 years. Results of histopathological examination showed chronic nonspecific inflammation in 28 patients (46%), features suggesting tubercular infection in 19 patients (31%), pigmented villonodular synovitis in seven patients (11.5%), rheumatoid arthritis in three (5%) patients, acute inflammation in three (5%) patients and findings suggestive of synovial chondromatosis in one (1.5%) patient. Treatment was based on histopathological results. Intra-articular injections of methylprednisolone (80 mg depot preparation) were given to all patients with nonspecific synovitis and rheumatoid arthritis. Anti-tubercular treatment was started for patients with tubercular synovitis. Complete arthroscopic/open synovectomy followed by radiotherapy was carried out for patients with pigmented villonodular synovitis. Non-steroidal anti-inflammatory drugs are used for patients with acute on chronic inflammation. All patients had symptomatic relief and functional improvement in further follow-up. *Discussion*: Histopathological reporting remains the mainstay for diagnosis. The various differentials should always be kept in mind when approaching patients with chronic mono-articular synovitis. Specific treatment can be started once the diagnosis is confirmed.

## Introduction

Synovium is the most metabolically active structure inside a joint and is involved very early in different disorders involving the joint. The knee joint has the largest reflections of synovial lining and is the most commonly involved joint in synovial disorders [1]. Common chronic conditions affecting the synovium include systemic inflammatory arthritis, infections, and local proliferative disorders. Chronic synovitis involving a single large joint remains a diagnostic dilemma because of equivocal results in laboratory and radiological investigations, and non-classical clinical presentation. Whereas a systemic condition such as inflammatory arthritis is likely to involve more than one joint, chronic infections and synovial disorders should be ruled out in mono-articular involvement. There is no established protocol in current literature to approach a case of monoarticular synovitis of the knee.

Physical, biochemical, and microbiological studies on synovial fluid have proven to be useful adjuncts for the diagnosis [2]. However, they have poor specificity and predictive values [3]. Synovial biopsy combined with synovial fluid examination has a better role in diagnosis [4–6]. Needle biopsy frequently fails to provide representative pathological tissue. Arthroscopic synovial biopsy is a simple, feasible, and useful way of obtaining representative pathological tissue [6]. Moreover, the joint can be visually inspected for a pattern of synovial proliferation. Additional procedures such as synovectomy or microfracture for cartilage defects can also be carried out at the same time if needed. Data on the relative prevalence of various diseases causing chronic mono-articular synovitis is lacking in the literature. Here, we aim to study the causes of chronic mono-articular synovitis of the knee joint, and its management. The study focuses on the diagnosis, management, and histopathological correlation.

## Material and methods

Sixty-one patients with unilateral knee swelling of more than six weeks’ duration, presenting to the outpatient department of our tertiary care hospital were prospectively enrolled in the study between July 2016 and September 2017. A detailed history (including fever for more than 2 weeks, loss of weight and appetite, malaise, previous history of tuberculosis) and clinical examination were noted. Informed consent was obtained from all of the patients. All patients underwent plain radiographs and magnetic resonance imaging (MRI) followed by arthroscopic examination with synovial biopsy. Erythrocyte sedimentation rate (ESR) and c-reactive protein were performed as serological markers of inflammation. The synovial fluid was sent for biochemical, cytological, and microbiological analysis. The biochemical examination included sugar and protein, the cytological analysis included cell count and cell type, the microbiological analysis included Gram staining and Ziehl–Neelsen staining, aerobic culture, and cartridge-based nucleic acid amplification test (CBNAAT) for tuberculosis.

**Figure 1:**
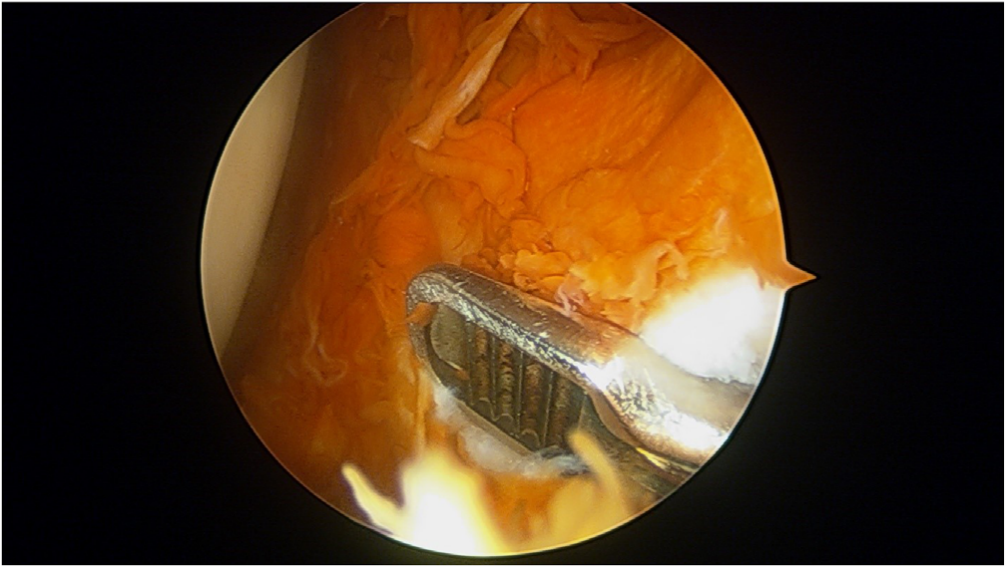
Intraoperative arthroscopic image showing punch biopsy being taken in a case of pigmented vilo-nodular synovitis.

We excluded patients with oligoarticular or polyarticular involvement, previously diagnosed or treated disease, and advanced osteoarthritis knee (Kellgren Lawrence grade 3 or 4). Diagnostic arthroscopy was done using standard anterolateral and anteromedial portals. Synovial tissue for biopsy was harvested from at least six sites within the joint including the suprapatellar pouch, medial and lateral gutter, the intercondylar notch, anterior and posterior compartments (Figure 1). Synovectomy was carried out and the joint was inspected for associated chondral changes or any other concomitant pathologies. Micro-fracture was done for localized chondral defects. Patients were allowed a full range of motion exercises and weight-bearing after the surgery (except when microfracture was performed, where the non-weight bearing protocol for six weeks was used).

**Figure 2:**
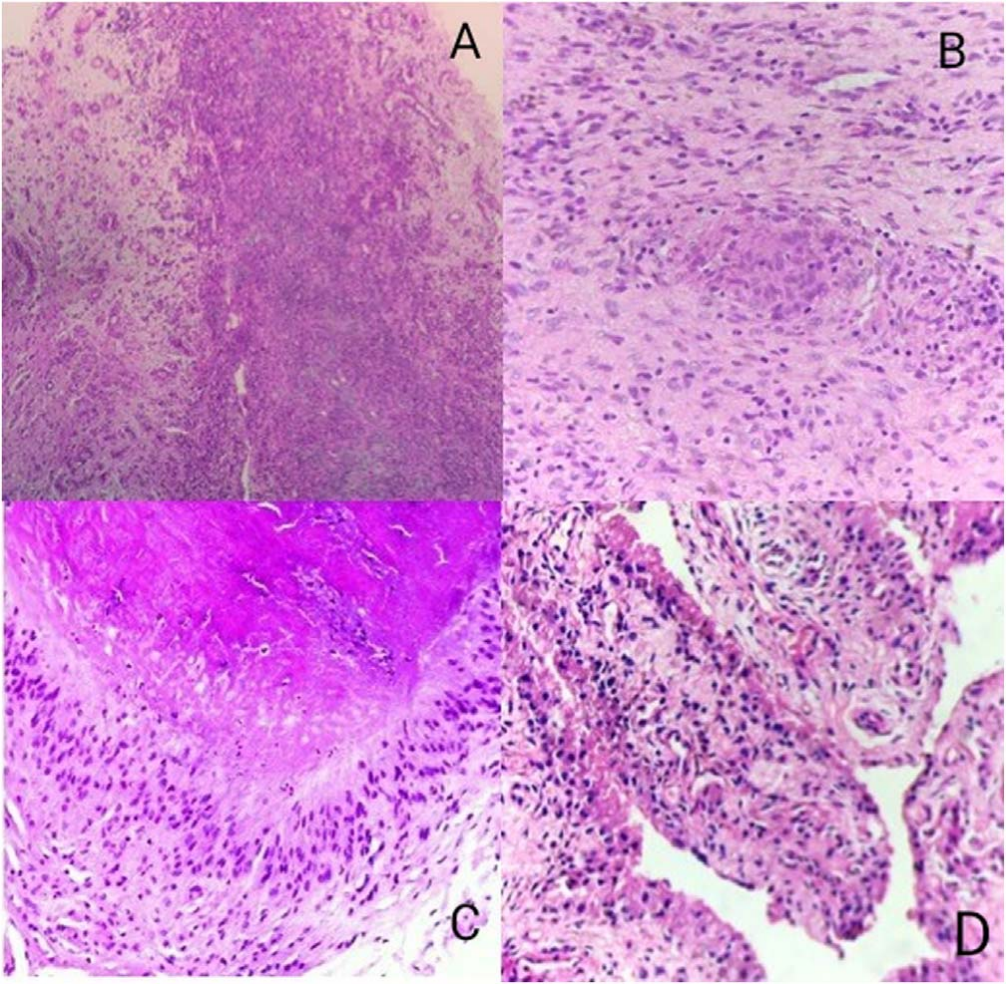
Figure depicting the 40× microscopic views of various etiologies of monoarticular synovitis of the knee (A – chronic synovitis, B – tubercular granulomatous inflammation, C – rheumatoid arthritis and D – pigmented villo-nodular synovitis of knee).

Clinical, radiological, and histopathological findings (Figure 2) were collaborated to make a final diagnosis. Patients were started on treatment accordingly. All patients were followed up with routine serological and radiological investigations until remission or up to two years from surgery. We assessed patients for knee swelling, range of motion and knee function using the visual analog scale (VAS) for pain and Western Ontario and McMaster Universities Osteoarthritis Index (WOMAC) score, at the presentation and at two-years follow-up.

### Statistical analysis

Statistical analysis was done using SPSS 24.0. Data was found to be normally distributed. Continuous variables are expressed as mean ± 2*SD*. A paired t-test was used to assess the mean difference between functional score, Visual Analog Scale, WOMAC score, and range of motion at presentation and 2-years follow up.

## Results

The mean duration of symptoms was 7.72 ± 4.34 months. The mean age at presentation was 29.93 years (13–43 years). There were 36 (59%) males and 25 (41%) females. Socioeconomic strata, according to Modified Kuppuswamy Scale [7] was upper-middle in 34 patients, lower-middle in 16 patients, and lower in 11 patients. The mean body mass index was 24.8 ± 4.6. Patellar tap and fluctuations were positive in all patients. Local tenderness and signs of synovial thickening were present in all the cases. None of the patients had constitutional symptoms.

**Figure 3:**
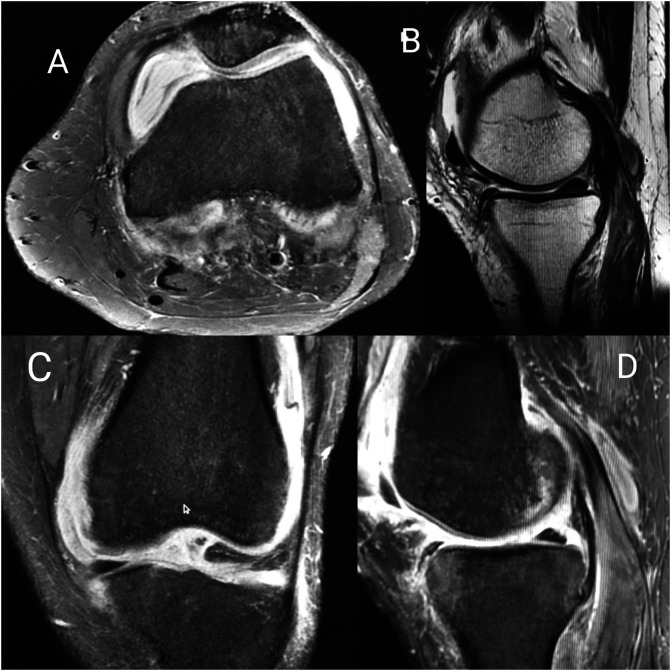
Figure showing MRI sections of a patient with inflammatory synovitis (A – axial PDFS, B – sagittal T2, C – coronal PDFS, sagittal PDFS showing hyperintensity in subarticular marrow of the tibia and femur with post-contrast enhancement and moderate effusion with diffuse enhancing synovial thickening).

**Figure 4:**
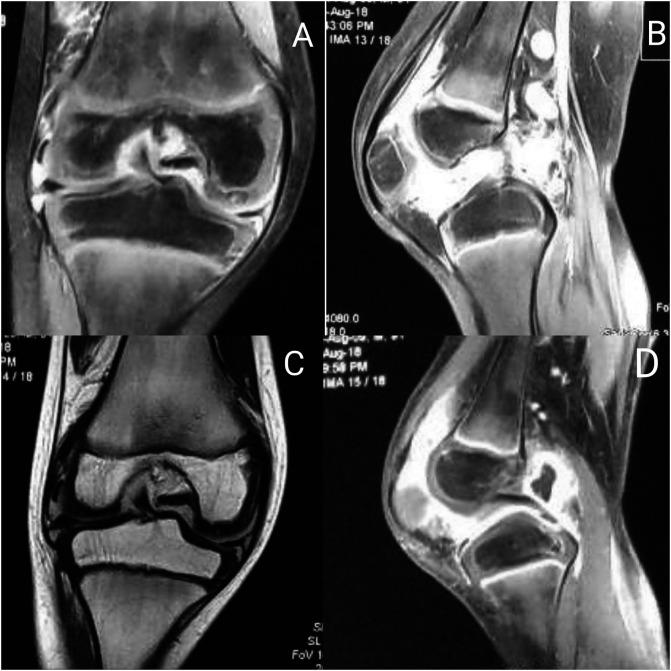
Figure showing MRI sections of a patient with infective pathology (A – coronal STIR, B – sagittal STIR, C – coronal T1, D – sagittal STIR images showing moderate effusion with peripherally enhanced intensities in popliteal fossa).

**Figure 5:**
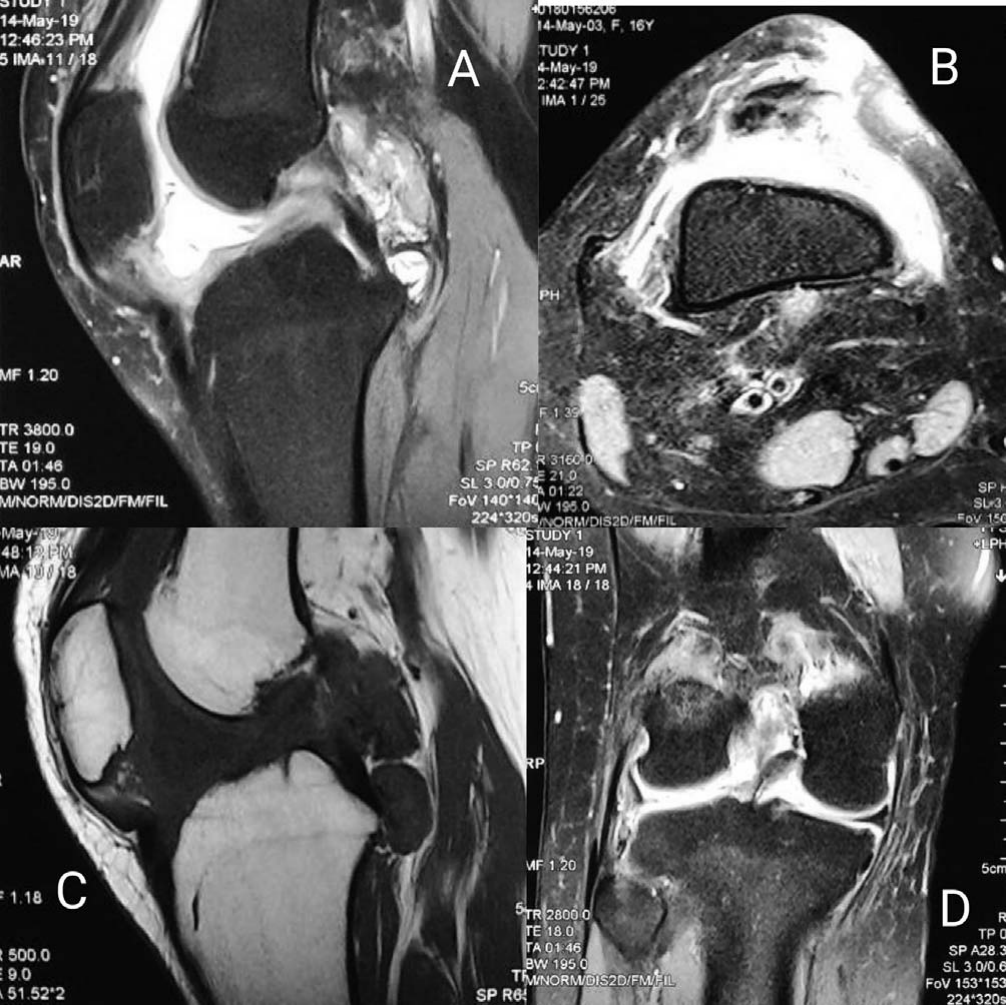
Figure showing MRI sections of a patient with pigmented villo-nodular synovitis of knee (A – sagittal STIR, B – axial STIR, C – sagittal T1, D – coronal STIR images showing nodular synovial thickening with foci of blooming within patellar tendon, Hoffa’s pad of fat, quadriceps tendon, and popliteal fossa).

Plain radiographs did not show signs of bone or cartilage loss in any patient. MRI showed synovial hypertrophy with or without effusion and juxta-articular bone marrow edema suggestive of inflammatory synovitis in 27 cases (Table 2) (Figure 3). In contrast-enhanced MRI (CE-MRI) relatively thin synovium with areas of peripheral rim, enhancements were noted in 11 patients, suggestive of infective etiology of knee synovitis (Figure 4). Seven patients had a low signal on both T1 and T2 weighted images suggestive of pigmented villonodular synovitis (Figure 5). MRI appearance suggestive of synovial chondromatosis as a differential was reported in one patient. Synovial fluid aspiration revealed yellow cloudy fluid suggestive of inflammatory pathology in 34 patients, yellow translucent fluid suggestive of non-inflammatory pathology in six patients, straw-like turbid low viscosity fluid with white blood cell counts suggestive of tubercular pathology found in 14 patients (Table 1). Reddish-brown synovial fluid was aspirated from seven patients which can be seen in post-traumatic hemarthrosis, pigmented vilo-nodular synovitis (PVNS) or hemophilic arthritis. Synovial fluid culture or Ziehl–Neelsen staining was not positive in any of the cases. Diagnostic arthroscopy revealed useful clues leading us to the diagnosis of seven PVNS cases. Inflamed, edematous synovial villi formation was observed in five patients suggestive of acute inflammation. However, the rest of the cases (*n* = 49) showed nonuniform findings of hypertrophied synovium with mild to moderate inflammation suggestive of inflammatory or chronic infective pathology. Etiological diagnosis based on MRI, arthroscopy, synovial fluid analysis, and synovial biopsy-based diagnosis has been shown in Table 2.

**Table 1:** Physical and cytological characteristics of synovial fluid.

	Colour	Clarity	White blood cell count (cells/mm^3^)	Staining
Normal	Colourless	Translucent	<200	
Non-inflammatory	Straw like/yellow	Translucent	200–2000	
Inflammatory	Yellow	Cloudy	2000–50,000 (>50% neutrophil)	
Septic	Yellow	Cloudy	>50,000 (>75% neutrophil)	Gram stain may be positive
Tubercular	Straw like	Cloudy/translucent	8000–12,000 (>50% neutrophil, >10% lymphocytes)	ZN stain may be positive

**Table 2:** Tabulation of diagnosis by MRI, arthroscopy, synovial fluid analysis, and synovial biopsy: results expressed as the number of cases (percentage).

Diagnosis	Etiologies (*n*, percentage [%])					
MRI findings/diagnosis	Inflammatory (27, 44%)	Infective (11, 18%)	PVNS (7, 11.5%)	Inconclusive infective/inflammatory (15, 25%)	Synovial chondromatosis (1, 1.5%)	
Arthroscopic findings	Infective/inflammatory (49, 80.5%)	PVNS (7, 11.5%)	Acute inflammation (5, 8%)			
Synovial fluid analysis	Inflammatory (34, 56%)	Tubercular (14, 23%)	Haemorrhagic (7, 11.5%)	Non-inflammatory (6, 9.5%)		
Synovial biopsy	Chronic nonspecific synovitis (28, 46%)	Necrotizing granulomatous inflammation (19, 31%)	PVNS (7, 11.5%)	Rheumatoid arthritis (3, 5%)	Acute inflammation (3, 5%)	Synovial chondromatosis (1, 1.5%)

*n* = number of cases.

Outerbridge grade 2 to grade 3 changes needing microfracture were noted in femoral and tibial articular surfaces in eight patients. Partial meniscectomy for degenerative tears of the medial meniscus was performed in three cases. Hypertrophied, inflamed synovium noted in all the patients in diagnostic arthroscopy. The cases of suspected PVNS showed diffusely inflamed synovium with brownish villi. Complete synovectomy was done in these cases. Histopathological and CBNAAT results have been summarized in Table 3.

**Table 3:** Comparison between histopathology and CBNAAT results.

Histopathological diagnosis	No. of patients (%)	CBNAAT positive
Chronic non-specific synovitis	28 (46)	–
Tuberculosis	19 (31)	17
Pigmented villonodular synovitis	7 (11.5)	–
Rheumatoid arthritis	3 (5)	–
Acute inflammation	3 (5)	–
Synovial chondromatosis	1 (1.5)	

Treatment decisions were based on histopathological results. Cases of chronic non-specific synovitis of the knee received intraarticular injections of 2 mL methylprednisolone acetate suspension (40 mg/mL) diluted with 10 mL normal saline. The injection was repeated after three months in cases of recurrence of symptoms. Anti-tubercular treatment (ATT) was started in patients where histopathology was suggestive of tubercular infection. The total duration of ATT was nine months. Complete synovectomy followed by radiotherapy was done for patients with pigmented villonodular synovitis. Anti-rheumatoid treatment was started after serological investigations in the rheumatoid arthritis group, and nonsteroidal anti-inflammatory drugs were given for patients with acute on chronic inflammation. All patients were followed up with complete blood count, inflammatory markers like erythrocyte sedimentation rate (ESR), C-reactive protein (CRPH) every three months until remission or up to two years. The tubercular synovitis cases were screened by liver function tests every three months. The prognosis of the patients turned out to be excellent in all of the cases of chronic nonspecific synovitis and acute inflammation except for three patients who had a recurrence of swelling within two years. Fixed flexion deformities ranging from 5° to 20° were noted in nine cases of tubercular monoarticular synovitis. Nevertheless, all of them had satisfactory pain relief and improved function at two years follow-up. Similar satisfactory progress was noted in patients with PVNS. However, two of seven PVNS patients had persistent swelling at 2-years follow-up. All patients in the rheumatoid group went into remission except in one patient who deteriorated to polyarticular joint involvement within the 2-years follow-up period. Visual Analog Scale, WOMAC score, and range of motion, noted at the presentation and two years follow-up have been summarized in Table 4.

**Table 4:** Analysis of VAS, WOMAC, and range of motion of all patients.

	At presentation			At 2 years			Mean improvement	*P*-value
	Mean	Standard deviation	Range	Mean	Standard deviation	Range		
VAS score	6.3	1.3	3–9	1.8	1.4	0–5	4.5 ± 1.7	<0.001
WOMAC score	61.4	14.4	38–86	23.6	9.6	11–44	37.8 ± 10.9	<0.001
Range of motion	82.4	28.1	25–120	115.6	16.3	60–130	33.2 ± 19.2	<0.001

## Discussion

Chronic monoarticular synovitis can result from a variety of conditions such as inflammatory arthritis, post-traumatic arthritis, degenerative arthritis, chronic infection such as tuberculosis, and synovial proliferative disorders. Difficulty in diagnosis often leads to indiscriminate use of steroidal and non-steroidal anti-inflammatory drugs for empirical treatment and symptom control. Whereas the utility of synovial biopsy in patients with polyarticular synovitis is questionable, patients with chronic monoarticular synovitis need histopathological examination for definite diagnosis [8–10]. Conventional laboratory studies and synovial fluid examination are a useful adjunct but not definitive for diagnosis. Needle biopsy has failed to provide enough representative samples of synovium and arthroscopic biopsy is generally recommended [6, 11, 12].

Chronic nonspecific synovitis was the most common (46.7%) histopathological finding in our series of patients. Differentiation between chronic inflammatory, infective, and proliferative conditions is an important distinction to be made and treatment strategy is widely different. Thick edematous, polyp-like, or club-shaped, synovium can be seen in inflammatory arthritis [13]. Prominent vascular networks with no thickening or villi formation can be seen in reactive arthritis. But the appearance of synovium is different in different stages of arthritis and the distinction between different types of inflammatory arthritis or differentiation from other causes of synovitis may not be always possible [13, 14]. This may be the reason for many cases being diagnosed as chronic non-specific synovitis.

Tubercular synovitis was relatively common (32%). This is similar to a study by Abhyankar et al. [15] (42.5%) and Singhal et al. [6] (26%). All 17 patients with a positive CBNAAT result had a histopathological examination suggestive of tuberculosis. Thus, CBNAAT was found to be 89.4% sensitive and 100% specific for the diagnosis of tubercular synovitis. Prominent MRI features were synovial hypertrophy, joint effusions, and post-contrast enhancement. Histopathological examination is most useful in diagnosis. Granulomatous inflammation with prominent epithelioid cells and Langerhans’ giant cells with or without caseous necrosis is seen on histopathology.

Hemosiderin deposition in the joint appearing as T1/T2 hypointense deposits along with synovial hypertrophy gives an important indication of PNVS, and MRI is a sensitive non-invasive diagnostic tool for these cases of synovitis. Treatment for chronic synovitis by arthroscopic or open synovectomy has always remained debatable. Literature suggests that arthroscopic synovectomy better than open synovectomy [14, 16]. The advantages of a minimally invasive procedure concluded in the study by Pan et al. like faster recovery, less post-operative pain, and better cosmesis suggest that arthroscopic procedure is better than its open counterpart, which is consistent with our study [17].

Correlation of arthroscopic diagnosis with the histopathological diagnosis has been established in cases of rheumatoid synovitis based on cartilage defects according to the American College of Rheumatology (ACR)/Knee Arthroscopy Osteoarthritis Scale [13]. However, it is difficult to differentiate between different stages of chronic infective and inflammatory pathologies and an objective classification system describing grades of changes in the synovium is still lacking in the literature [18, 19]. Thus, we could only differentiate PVNS (*n* = 7) and acute inflammatory (*n* = 4) cases from others based on arthroscopy.

Based on clinic-radiological, arthroscopic, and histopathological evidence we reached the final diagnosis in all of our cases, which was comparable to previous studies by Singhal et al. [6], Balireddy et al. [12] Chen et al. [20] (Table 5). Compared to these studies [6, 12, 20] the present study highlights a relatively higher number of cases of PVNS (*n* = 7, 12%) while there were no cases of monoarticular knee gouty arthritis.

**Table 5:** Comparison of results of histopathological diagnosis to other studies in the literature.

Pathology	Singhal et al. [6] (*n* = 50)	Balireddy et al. [12] (*n* = 40)	Chen et al. [20] (*n* = 74)	Our study (*n* = 61)
Nonspecific synovitis	10 (20%)	25 (62%)	–	28 (46%)
Tuberculous synovitis	13 (26%)	7 (18%)	5 (6.5%)	19 (31%)
Rheumatoid synovitis	14 (28%)	6 (15%)	39 (52.9%)	3 (5%)
Septic arthritis	3 (6%)	–	7 (9.5%)	–
Pigmented villonodular synovitis	1 (2%)	1 (2.5%)	1 (1.3%)	7 (11.5%)
Gouty synovitis	2 (4%)	1 (2.5%)	11 (14.6%)	–
Synovial Chondromatosis	–	–	–	1 (1.5%)
Seronegative spondyloarthropathy	–	–	7 (9.5%)	–
Multicentric reticulohistiocytosis	–	–	1 (1.3%)	–
Acute inflammation	–	–	–	3 (5%)
Osteoarthritis	3 (6%)	–	–	–
Post traumatic	4 (8%)	–		–
Undiagnosed	–	–	3 (4.4%)	–

Intra-articular injection of methylprednisolone has proven to be effective in cases of rheumatoid arthritis in multiple clinical trials [21, 22]. Optimal use has been shown to improve both pain and mobility in cases of inflammatory synovitis [23, 24]. However, the utility of local steroids in cases of chronic non-specific synovitis is yet to be understood. In our study, chronic nonspecific synovitis cases showed excellent results after treatment with intra-articular steroid following synovectomy, in terms of pain, functional scores, and range of motion except for the cases (*n* = 3) of recurrences (Table 6). The high recurrence rate reported in the literature in cases of non-specific synovitis makes it questionable to treat these cases by only arthroscopic synovectomy [11]. The addition of radiosynoviorthesis as an adjunct to synovectomy has shown promising results in some studies [25, 26]. Karaman et al. [26] demonstrated arthroscopic synovectomy and radiosynoviorthesis to have better functional outcomes than either of those treatments used alone. However, there is a need for strong evidence to prove its superiority over other treatment methods.

**Table 6:** Comparison of outcome parameters of different diseases.

Diagnosis	Mean improvement (standard deviation)			*P*-value		
	ROM	WOMAC	VAS	ROM	WOMAC	VAS
Chronic non-specific synovitis	20.9 (12.2)	31.1 (10.6)	3.9 (1.4)	<0.001	<0.001	<0.001
Tubercular synovitis	52.4 (13.2)	45.6 (5.8)	5.5 (1.6)	0.01	0.01	0.01
PVNS	28.5 (10.7)	37.4 (9.7)	4.1 (1.2)	0.01	0.01	0.01

**Figure 6:**
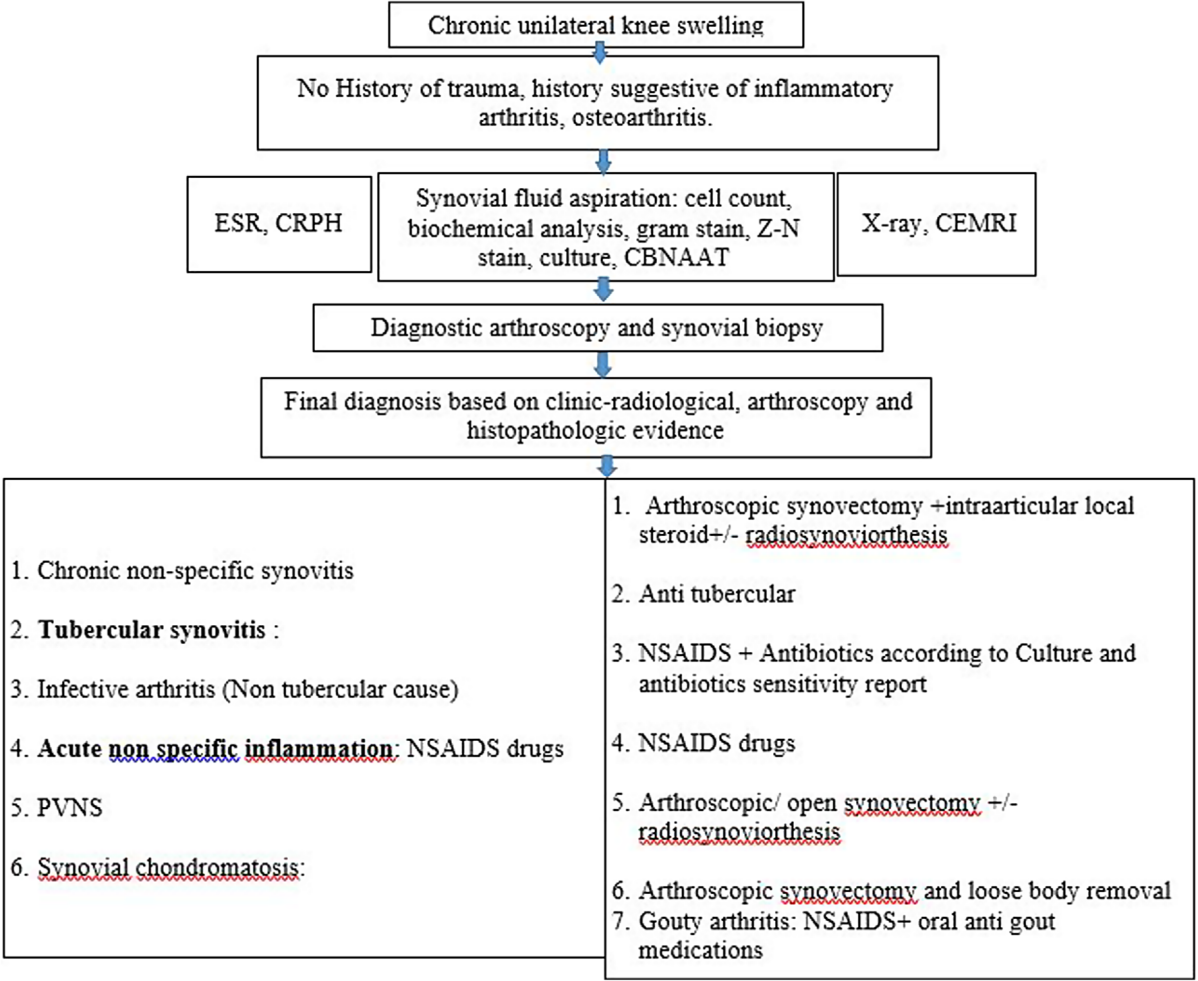
Flowchart showing treatment plan in chronic monoarticular synovitis.

Satisfactory results were observed in cases of tubercular synovitis with appropriate ATT started at the correct time. Two of the patients had ATT induced hepatitis which was managed by a modification of the ATT regimen. No other patient had any treatment-related complications in other groups. Two of the patients among the PVNS group and three patients from the chronic nonspecific synovitis group had persistent swelling at two-year follow-up. These patients had an overall unsatisfactory result and were unhappy with the treatment process. In comparison with the tubercular synovitis group, patients with chronic nonspecific synovitis had significantly better functional outcomes (mean difference: 11.34, *p* < 0.01) and range of motion (mean difference: 20.9, *p* < 0.01). The current observational study focused not only on the diagnosis of chronic monoarticular synovitis but also on the treatment approach. A flowchart illustrating the treatment approach to any patient with monoarticular synovitis in the knee is shown in Figure 6. Despite the superiority over laboratory investigations and synovial fluid analysis, many a time no specific diagnosis can be made even after the synovial biopsy leading to a diagnosis of chronic non-specific synovitis. This gives rise to a diagnostic and treatment dilemma, which can be addressed with intra-articular steroids and NSAIDs thereafter. Despite a short follow up, we had remission of disease in most of the cases in our study. This study takes us a valuable step forwards towards the diagnosis and management of chronic monoarticular synovitis.

## Conclusion

Histopathological reporting remains the mainstay for diagnosis of chronic monoarticular synovitis. The various differentials like tubercular synovitis, PVNS, monoarticular rheumatoid should always be kept in mind when approaching these patients. Arthroscopic synovial biopsy can be useful and productive for early diagnosis. Specific treatment can only be started for management once the diagnosis is confirmed. Patients need to be followed up with necessary blood parameters and feasible physiotherapy protocol until remission. Intraarticular steroid injection following synovectomy for chronic nonspecific synovitis may be a definitive treatment modality.

## Limitations

Our prospective study has certain limitations like short follow up and smaller sample size. The histopathological examination has its own limitations. It may be difficult to differentiate between early rheumatoid arthritis and non-rheumatoid synovitis [27].

## Declaration of conflicting interests

The author(s) declared no potential conflicts of interest with respect to the research, authorship, and/or publication of this article.
